# Gastroschisis: diagnosis, prognosis and treatment options

**DOI:** 10.3389/fped.2025.1717874

**Published:** 2025-12-02

**Authors:** Dastan Rustemov, Zhenis Sakuov, Zhuldyz Kucherbayeva, Sherkhan Shayakhmetov, Ruslan Bilal

**Affiliations:** 1 Department of Pediatric Surgery, University Medical Center, Mother and Child Center, Astana, Kazakhstan; 2 Department of Surgery, School of Medicine, Nazarbayev University, Astana, Kazakhstan

**Keywords:** gastroschisis, neonatal surgery, congenital abdominal wall defects, prognosis, surgical treatment, complications, siloplasty, surgery

## Abstract

Gastroschisis is a rare congenital anomaly characterized by extrusion of abdominal organs through a defect in the anterior abdominal wall, typically to the right of the umbilical cord. Despite advances in prenatal diagnostics and neonatal surgical care, gastroschisis remains associated with significant morbidity and mortality. This review summarizes recent epidemiological data, etiological hypotheses, diagnostic approaches, treatment strategies, and postoperative complications. Both simple and complicated forms are discussed, with emphasis on the impact of viscero-abdominal disproportion and associated anomalies. Recent modifications in surgical tactics, including staged reduction and innovative siloplasty techniques, are evaluated. The review highlights ongoing challenges in optimizing surgical timing, minimizing complications such as adhesive disease and necrotizing enterocolitis, and improving long-term outcomes.

## Introduction

Gastroschisis is a congenital malformation of the anterolateral abdominal wall. Over the past 10 years, the incidence of this malformation has been 5–6 per 10,000 live births. Here are some other data: 1.75: 10,000 according to C. Stoll et al. for the period 1979–1998 (observations were conducted in Europe, primarily in France), 1.54: 10,000 according to a multicenter study for 1996–1998 (observations were conducted in 11 European countries) (1).

It occurs with approximately equal frequency in boys and girls (although, according to some data, with a slight predominance among boys). Approximately 80% of patients with gastroschisis are premature (2), born on average at 35–37 weeks of pregnancy) and have prenatal hypotrophy (the average birth weight does not exceed 3,000 grams) (3). It was also noted that newborns with gastroschisis are mainly born to mothers under 24 years of age (4). If the mother was 15–19 years old, the incidence of gastroschisis in newborns increased sharply compared to the general population and was at the level of 26.4; 10,000 people. It is also interesting that in repeated pregnancies, the incidence of gastroschisis in the fetus is at the level of 5%–6% (5).

A number of authors note such features of the mother's diet as a lack of glutathione, carotenes, high content of nitrosoin, which may be associated with an increased risk of developing gastroschisis in the fetus (6). Finally, taking non-selective cyclooxygenase inhibitors can also lead to the development of gastroschisis due to the suppression of prostacyclin formation in endothelial cells (7). The mortality rate of patients with gastroschisis remains high, fluctuating quite significantly within the range from 6.4% to 47% depending on the expertise of the medical center, the region, and the presence of complications. In leading pediatric surgery centers in the USA and Western Europe, the mortality rate does not exceed 17% (8).

### Etiology and pathogenesis of gastroschisis

From the point of view of pathological anatomy, this defect is characterized by an intrauterine defect of the anterior abdominal wall, most often this defect is located to the right of the normally formed umbilical cord, through which the abdominal organs are eventrated (9).

Gastroschisis was first studied in the 1920s. The first works published on this topic belong to Klopp et al., 1921; Delaglade et al., 1928; Graser et al., 1929. Then, for the first time, it was suggested that the etiology of the development of the defect of the anterior abdominal wall is associated with an intrauterine rupture of the membranes of the formed umbilical cord hernia (10).

However, starting around 1940, it was proven that gastroschisis is an independent developmental defect and is not associated with the formation of an umbilical cord hernia (11).

Currently, the vascular theory of gastroschisis remains the most convincing (12). A large number of pathomorphological observations have shown that gastroschisis is combined with aplasia or early involution of one of the umbilical veins (usually the right one). There are studies in which thrombosis of the right umbilical vein was observed with a normally formed right umbilical vein (13).

Finally, a number of studies have shown that gastroschisis was associated with early involution of the right omphalomesenteric artery (14).

Ischemia of the above-mentioned vessels carrying oxygenated blood most likely disrupted the trophism of embryonic tissues, which led to the development of ecto- and mesodermal defects, disruption of the formation of the terminal segment of the omphalomesenteric artery feeding the right mesogastric region (15).

Pathomorphology of the defect, its place in the dysembryogenesis of the anterolateral abdominal wall, combination with other defects of the intestinal tube.

Diagnosis of the developmental defect of the anterior abdominal wall does not cause difficulties. In gastroschisis, eventrated intestinal loops are clearly visible, most often—the small and large intestine, always on the common mesentery with a fairly narrow root, the width of which corresponds to the thickness of the defect of the abdominal wall (16).

Of particular interest is a rare variant termed closed gastroschisis, characterized by an initial herniation of the intestines through the abdominal wall defect subsequently leads to spontaneous defect closure during fetal development before the intestines return to the abdomen. This condition is also known as vanishing midgut syndrome, which refers to a rare and severe condition where the midgut, which includes a significant portion of the intestines, undergoes ischemic injury, leading to extensive resorption or atrophy. This condition can result in substantial loss of bowel length, leaving the foetus with a markedly shortened or absent midgut (17).

## Methods

A comprehensive literature search was conducted in PubMed, Web of Science, Google Scholar, and eLibrary, using the terms *gastroschisis, neonatal surgery, abdominal wall defects, prognosis, surgical intervention*. Articles in English and Russian from the last 10 years were reviewed. Over 100 publications were analyzed to synthesize current evidence on diagnosis, management, and outcomes of gastroschisis.

## Results

### Diagnosis

Diagnosis of gastroschisis is possible (undoubtedly) in the prenatal period, starting from the 11th week of intrauterine development (the optimal period for ultrasound screening is 10–14 weeks of gestation). The frequency of “missed” diagnoses is extremely low and is possible only in cases where Doppler mapping is not used and intestinal loops are mistaken for the umbilical cord (18).

An additional diagnostic method (starting from the second trimester of pregnancy) is a quantitative analysis of the mother's blood serum for alpha-fetoprotein (19).

It is worth noting that at present this pathology is completely curable, and therefore this diagnosis is not an indication for termination of pregnancy (20).

The criteria for gastroschisis are:

1. A correctly formed umbilical cord (21)
2. The absence of walls of the hernial sac or limiting membrane (22)
3. The location of the defect of the anterolateral abdominal wall outside the umbilical ring (23)

The organs of the lower floor of the peritoneum are eventrated quite rarely; in boys, undescended testicles may be eventrated, and in girls, the appendages (24). The liver is always located in the abdominal cavity, is correctly formed, and is fixed in the right hypochondrium by the falciform ligament (25). All eventrated organs are covered with fibrin films. The stomach and intestines are usually enlarged in size and have weak peristalsis. The walls of hollow organs are usually infiltrated (26).

A false shortening of the intestinal tube is noted (on average by a third), in a fibrin “case” it is as if corrugated. With skeletonization of intestinal loops (or natural resorption in the postnatal period), the length of the intestinal tube is most often normal (27). In gastroschisis, in contrast to other congenital defects of the anterolateral abdominal wall (for example, umbilical ring hernia), combined defects are extremely rare. The literature describes such combined defects as atresia of the small/large intestine, Meckel's diverticulum (28). Extraintestinal anomalies associated with gastroschisis occur in 6% of cases, such anomalies as aplasia of the ureter and ureterohydronephrosis, renal aplasia are described. Only 1% of patients with gastroschisis have congenital heart defects and central nervous system defects, which does not distinguish this cohort of patients from the general population and is probably random (29).

### Classification

Gastroschisis is usually classified into 2 forms—simple and complicated. The simple form of gastroschisis is characterized by the presence of an isolated defect, usually without visceroabdominal disproportion (up to 80%)—a discrepancy between the volume of visceral organs and the conditional volume of the abdominal cavity, formed when the defect is closed without additional plastic interventions (30).

The complicated form of gastroschisis is characterized by the presence of combined malformations and prenatal complications. Visceroabdominal disproportion occurs in up to 20% (31).

The division of gastroschisis into simple and complicated is dictated by different surgical tactics and different postoperative results (32).

It is worth noting separately that there is a so-called “closed form of gastroschisis”. Anatomically, it is expressed in the presence of a narrow defect in the anterior abdominal wall. The walls of the defect often compress the eventrated organs, which leads to the development of tissue ischemia. The conglomerate of eventrated organs is covered with a dense fibrin film, due to which the separation and skeletonization of organs is impossible (33).

This form is considered as intermediate between simple and complicated, surgical tactics involve refusal of one-stage correction of the defect, immersion of the conglomerate of visceral organs into the abdominal cavity in the hope of fibrin resorption, application of an enterostomy for intestinal decompression, delayed revision of the abdominal cavity after 1–1.5 months. “Closed” gastroschisis is quite rare and usually has a favorable outcome—the frequency of successful organ-preserving interventions is at the level of 70%–76% (34).

### Perinatal and prehospital care

At the prehospital stage, primary resuscitation measures for a patient with gastroschisis are provided by a neonatal team, which should include a pediatric resuscitator and a surgeon. The most important task of patient care at the prehospital stage is maintaining normal body temperature, preventing hypothermia due to pronounced heat loss through the eventrated organs (35).

Also, priority tasks include gastric decompression (in order to reduce visceral-abdominal disproportion) and prevention of aspiration (nasogastric intubation is provided for this purpose), gastric lavage, high colon lavage with a solution containing acetylcysteine and pancreatin suspension, adequate analgesia (using non-narcotic analgesics in age-appropriate doses), replenishment of circulating blood volume with crystalloid solutions, use of inotropic support in case of a tendency to hypotension, and transfer of the patient to “controlled breathing”. Then, as soon as possible, the patient is transported to a surgical hospital in order to determine further treatment tactics (12).

### Surgical treatment

The surgical tactics of patient management also differ depending on the form of the congenital malformation. Thus, simple forms of gastroschisis are usually corrected simultaneously and radically, the survival rate after one-stage correction of the abdominal wall defect tends to 100%, in relation to complicated forms, staged surgical interventions are mainly used, and the possibilities of reconstruction are limited by the accompanying pathology (36).

Surgical treatment of gastroschisis dates back to 1951, when Moore et al. adapted the Gross operation, 1948 (developed for the correction of extensive umbilical cord hernias) for the treatment of gastroschisis (37).

Accordingly, historically, the first method of correction was primary radical plastic surgery of the abdominal wall defect (38).

The essence of the Moore operation was the formation of a ventral hernia by separating and mobilizing skin flaps—primary plastic surgery with a skin flap with delayed surgery for a ventral hernia. Mortality after this intervention, according to the results of a series of 36 clinical cases, was at the level of 70% (39).

The unsatisfactory result was associated with severe compression of the internal organs of the abdominal cavity. In subsequent years, this operation was supplemented by resection of several intestinal loops to overcome the visceroabdominal disproportion. Later, there were reports on the formation of unloading gastrostomy, enterostomy, some authors also suggested applying pneumoperitoneum. Certain importance was attached to manual stretching of the muscles of the anterior abdominal wall in order to increase the volume of the abdominal cavity (40).

A less radical method of primary radical correction is the Bianchi procedure—“bloodless” and “anesthetic-free” reduction of eventrated organs (41).

A separate direction of surgical correction has also formed, which can be attributed to the group of delayed radical plastic surgery. This group includes siloplasty and alloplasty. A new era in the treatment of gastroschisis began with the work of S. Schuster (1967), who proposed using a Teflon bag as a temporary container for visceral organs that do not fit into the abdominal cavity. The intervention was called “siloplasty”. The Teflon bag was sutured to the edges of the defect in the anterior abdominal wall (42).

### Complications

Complicated forms of gastroschisis significantly limit the possibilities of radical surgical treatment and require additional care. The presence of complications has a significant impact on treatment tactics, which sometimes leads to the need to use non-traditional methods and individual selection of the scope of intervention. Below, the most serious complications of gastroschisis are considered, and an analysis of the literature on the management of patients with these complications, with varying degrees of evidence, is conducted (43).

Complicated gastroschisis requires special attention and careful assessment before surgery, but in most cases radical closure of the abdominal wall defect remains appropriate. This is due to the fact that successful closure of the defect can significantly improve the patient's quality of life and prevent further complications. It is important that surgical tactics are based on individual indicators and the patient's condition, taking into account all the potential risks and benefits of the operation (44).

On the contrary, clinical cases of one-stage radical correction of gastroschisis have been described, which was combined with intestinal loop necrosis (resection with primary intestinal anastomosis), short bowel syndrome (in which one-stage gastrostomy was performed), intestinal atresia and stenosis (in such cases, most specialists recommend resection with primary intestinal anastomosis if it is possible to avoid tension between intestinal loops) (45). In general, according to many authors, early correction of complications, like early correction of gastroschisis, is prognostically associated with better postoperative results (46).

### Mesenteric vascular thrombosis

Mesenteric vascular thrombosis can be considered a serious postoperative complication, especially in patients with severe visceral-abdominal disproportion. This situation can arise during one-stage plastic surgery of a defect in patients with severe visceral-abdominal disproportion (47).

In the world literature, most cases of mesenteric artery thrombosis are associated with the implantation of an allogeneic flap, although it is quite difficult to trace the correlation of this complication with the plastic material (48).

In all cases of mesenteric thrombosis, revision of the abdominal organs is indicated, followed by assessment of the viability of the intestinal loops. Most often, subtotal resection of the small intestine with the imposition of a primary anastomosis is performed (49). Researchers from one of the European centers have shown that in the case of mesenteric thrombosis that develops after subtotal resection of the intestine, layer-by-layer suturing of the abdominal cavity is indicated if:

1. after resection of the intestine, the visceral-abdominal ratio was equalized (50).
2. “Non-violent” connection of the edges of the defect is possible and there are no signs of high tension at the suture site and/or signs of increased abdominal pressure (51)
3. the afferent and efferent loops of the intestine were recognized as viable and a sufficiently extensive resection was performed, taking into account the fact that the actual volume of intestinal damage is 20%–30% greater (52)
4. there is no diffuse peritonitis, there were no signs of peritonitis with sepsis (53).
5. there is no pronounced edema of the intestinal loops, which potentially carries the risk of disruption of the blood supply to the submucosa with subsequent increase in the permeability of the intestinal wall (37).

Researchers from another center also added that almost always at the time of revision laparotomy, signs of aseptic peritonitis with an exudative or fibroplastic reaction are observed in the abdominal cavity (38).

In these conditions, the tactic of suturing the peritoneum is optimal, since the mesothelium has the best protective, resorptive and barrier characteristics, due to which the inflammatory process under the peritoneal sheet is stopped more effectively, the incidence of adhesive disease is lower (39).

### Adhesive disease

Adhesive disease is one of the most common late postoperative complications in surgical correction of gastroschisis. Most often, this complication appears during the first year after plastic surgery of the anterior abdominal wall defect. The incidence of adhesive disease, according to some centers, reaches 18% and depends mainly on the plastic material used and the presence of infectious complications in the postoperative period. Thus, one study showed that infection of the skin suture after primary plastic surgery of the abdominal wall defect was accompanied by the postoperative development of adhesive disease (40). Severe tissue tension and high intra-abdominal pressure have a negative effect on tissue trophism and regeneration at the site of reconstruction, which increases the risk of peritonitis. The choice of plastic material should also be approached selectively (54). The use of synthetic materials, which are widely used in herniology, is most likely poorly suited for staged correction of gastroschisis, especially in cases where delayed suturing of the patient's own tissues with the removal of the initially implanted patch is planned (41). It should also be noted that adhesive disease often manifests itself in the form of adhesive intestinal obstruction. Its incidence is associated with the type of surgical intervention: for example, with the Bianchi operation it is 58.3%, while with surgical defect plastic surgery (siloplasty, Gross operation, alloplasty or plastic surgery using synthetic patches)—30.4%, according to a study conducted on a sample of 62 patients (55).

### Necrotizing enterocolitis

This complication is registered in approximately 20%–30% of cases of gastroschisis, is a significant factor in mortality, as well as early postoperative complications (56).

One of the retrospective studies examined predictors of the development of necrotizing enterocolitis in patients who underwent one-stage plastic surgery of the anterolateral abdominal wall defect. In a sample of 60 patients, 15% developed necrotizing enterocolitis. When comparing the groups (with and without this complication), it turned out that a statistically significant factor in the development of enterocolitis was low birth weight (57).

The authors of the study also emphasize the importance of nutritional support in reducing the risk of necrotizing enterocolitis. Feeding with expressed breast milk, in contrast to artificial feeding, significantly improved the prognosis regardless of birth weight and other factors (the presence of other complications, combined malformations) (58).

Further studies of the factors in the development of necrotic enterocolitis made it possible to formulate a concept according to which, with a confidence interval of more than 95%, this complication develops in patients with a combination of two or three factors (59):

1. an episode of intestinal ischemia due to compression in a defect in the abdominal wall or with abdominal compartment syndrome (if primary plastic surgery of the defect was performed with severe visceral-abdominal disproportion) (60).
2. colonization of the intestine with pathogenic bacteria
3. excess proteins in the intestinal lumen (with suboptimal artificial feeding)
4. Correction of extensive defects of the anterior abdominal wall

### Optimal timing

Considering that gastroschisis is rarely combined with any other congenital defects, the fetus is viable. Therefore, in most cases, it is recommended to continue the pregnancy. Early screening for gastroschisis is provided to optimize delivery and further monitoring (61).

Speaking about the optimal timing of treatment for patients with gastroschisis, it is worth touching on the issue of delivery. Until 2010, the concept of early delivery was quite popular in the scientific community. Early delivery was justified by the fact that it reduces the time of exposure of eventrated organs to amniotic fluid, which, in turn, reduces the risk of abdominal complications and reduces visceral-abdominal disproportion (62).

However, over time, a fairly large number of observations have proven that early delivery by cesarean section has a statistically insignificant effect on the outcomes of gastroschisis treatment compared to cesarean section at 30–32 weeks (63). Later, the results of studies were published, which showed that vaginal birth is more optimal in uncomplicated pregnancy, associated with less trauma to visceral organs than with cesarean section (64).

To this day, the following tactics for managing pregnancy and childbirth are accepted: if dynamic observation reveals a rapid increase in polyhydramnios or there are pathological changes in the eventrated organs, such as dilation of intestinal loops or severe edema, then early delivery by cesarean section is indicated, but if there are no such complications, then the pregnancy is recommended to be maintained, and childbirth should occur naturally (27).

Considering that newborns with gastroschisis have hypotrophy, the question of the timing of surgical intervention remains quite complex. At the same time, there is an opinion according to which surgical intervention is indicated as early as possible and in the presence of criteria for the patient's “readiness” for surgery.

Thus, Yu. I. Kucherov and co-authors suggest operating on a newborn in the first 5 h after birth, as this reduces the risk of infection of visceral organs and decompensation of homeostasis. The criteria for “readiness” are (65):

a. Stable peripheral and central hemodynamics
b. Satisfactory microcirculation
c. Diuresis more than 1 milliliter per 1,000 g of body weight per hour.

### Treatment options

The treatment tactics for patients with gastroschisis are determined by the anatomical possibility of immersion of eventrated organs into the abdominal cavity. Until now, the leading problem of correction has been increased intra-abdominal pressure, leading to the development of abdominal compartment syndrome.

This syndrome is characterized by the development of severe complications associated with increased intra-abdominal pressure. These complications affect central hemodynamics, for example, an increase in pressure in the abdominal cavity to 20 centimeters of water column can lead to compression of the inferior vena cava, which reduces venous return. In addition, compression of the mesenteric vessels and ischemia of intestinal loops are possible (66).

Intraoperative monitoring of intra-abdominal pressure is an important marker, based on which it is possible to determine the optimal volume of intervention. They resort to various indirect methods of monitoring intra-abdominal pressure, including measuring the pressure in the stomach, in the bladder, recording a tachogram, measuring the saturation of the tissues of the lower extremities.

The degree of tension of the tissues of the anterior abdominal wall is also visually assessed, but this method should not be used as the only one due to its inaccuracy (20).

In the presence of complicated forms of gastroschisis, the treatment tactics remain the same, however, the correction of life-threatening conditions comes to the fore. In this regard, it is often necessary to postpone plastic surgery of the abdominal wall defect with preliminary imposition of a temporary enterostomy (67).

### Radical correction of the defect

Most patients with gastroschisis undergo primary radical plastic surgery of the defect. Before this, it is necessary to return the eventrated visceral organs to the abdominal cavity, which is achieved by widening the abdominal wall defect. Among the various methods of immersion, the Bianchi method, which was mentioned earlier, deserves special attention. With this method, a gradual immersion of the visceral organs occurs without the use of general anesthesia (in some cases, and very rarely, epidural anesthesia is used) (68).

In the intensive care unit, in the presence of an anesthesiologist, high intestinal lavage is performed in order to reduce the volume of intestinal loops. After this, sutures are placed on the edges of the defect. Then traction is performed on the sutures—the sutures—the sutures—the sutures—as a result of which a reduced pressure is created in the abdominal cavity, as a result of which the eventrated organs are reduced (69).

The procedure can last for 40–60 min, while it is not recommended to perform separation of fibrin plaque. It is important to monitor intra-abdominal pressure using one of the monitoring methods discussed earlier (70).

After the defect has been reduced, defect plastic surgery is indicated, which is usually performed by layer-by-layer tissue connection in the transverse direction (71).

The advantage of this method is its simplicity, the ability to perform without connecting to an artificial lung ventilation device, early restoration of gastrointestinal tract function, and a reduction in the length of hospital stay (22).

However, it should be noted that this method has limitations, in particular, it cannot be used in the presence of stony infiltrate of visceral organs, the presence of a single dense conglomerate of eventrated organs, and it is also not used in the presence of complications, a high risk of infection of the abdominal cavity (72).

The time frame for this intervention is also quite strict, according to some data, it is recommended to perform the Bianchi procedure no later than 6 h after birth (61).

### Stage correction

If the patient has a viscero-abdominal disproportion, an attempt at one-stage radical correction of the defect is associated with unfavorable treatment results due to the development of increased intra-abdominal pressure syndrome (63).

Taking this into account, an operation is used to sew a patch into the abdominal wall defect that cannot be primarily sutured. The use of various materials as patches has been described. Among them are synthetic ones—GoreTex, MarTex, dacron mesh, allogeneic and xenogeneic biological materials—preserved dura mater, xenopericardium (73).

Of the above biological materials, the first to be used were synthetic ones, which were mainly developed for herniology and had the property of high adhesion to tissues. This made it difficult to perform a radical operation in the delayed period, since it was impossible to cosmetically remove the patch, and in addition, at the site of tension of the sutured tissues (and tension during such interventions almost always occurs, even if the intra-abdominal pressure is not exceeded), there was a risk of infection, after which severe paraprosthetic purulent complications developed. Therefore, at present, many use biological materials that are more resistant to infection and are easier to remove (29).

It is important to note that the patch is implanted only for a short period until the visceroabdominal disproportion is compensated. After this, its removal is indicated with the connection of the muscular-aponeurotic layers and the restoration of normal anatomy (74).

Such staged interventions allow the correction of gastroschisis to be “stretched” for 3–6 months, while reducing the risk of developing compartment syndrome (75). At the same time, this type of intervention is suboptimal due to the high risk of suture suppuration and the need to perform a full-fledged abdominal surgery using general anesthesia.

In this regard, in cases of severe disproportion, siloplasty is used. The procedure involves suturing a polymer bag to the edges of the defect (76). As a result, the eventrated visceral organs are in a closed space, as if being a continuation of the abdominal cavity. At the same time, sterility is maintained in the silo bag, the likelihood of contamination is reduced, and there is no loss of moisture (77).

The silastic bag allows for gradual reduction of visceral organs over 7–10 days, while there is the possibility of visual monitoring of the condition of the organs and their blood supply. It is important to gradually reduce the volume of the bag, exerting adequate pressure to immerse the eventrated organs (12). Upon completion of the immersion, the stability of the patient's somatic condition and tolerance of the existing intra-abdominal pressure are assessed. If the indicators are optimal, the bag is removed with subsequent correction of the defect. The limitation of siloplasty is the time of application of the polymer bag—if it is left in place for more than 10 days, there is an increased risk of infection at the points of contact of the synthetic material with the tissues of the anterior abdominal wall (78).

### Ways to increase freedom from early and delayed intra-abdominal complications in surgery for complicated forms of gastroschisis

**Figure 1:**
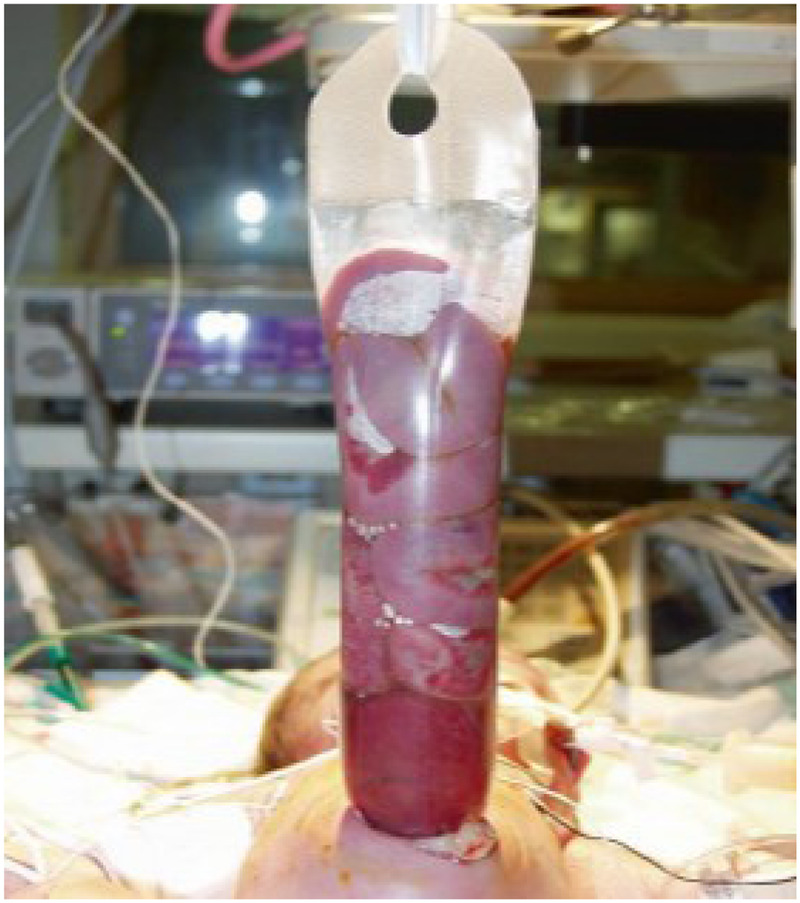
The patient with gastroschisis. Source: own photo.

**Figure 2:**
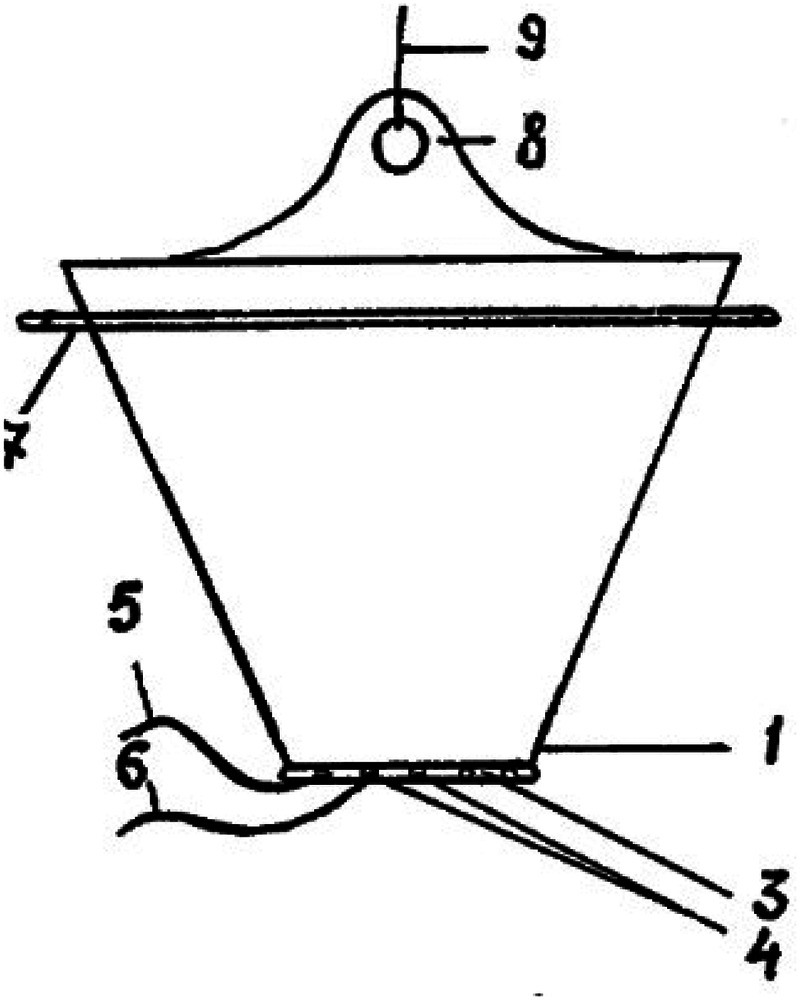
Comparative evaluation of the results of primary and delayed plastic surgery of the anterior abdominal wall using a new method in patients with gastroschisis. Source: https://cyberleninka.ru/article/n/sravnitelnaya-otsenka-rezultatov-pervichnoy-i-otsrochennoy-plastiki-peredney-bryushnoy-stenki-novym-sposobom-u-bolnyh-s-gastroshizisom.

In a study published in 2022, the authors examined the results of two tactics for treating gastroschisis—the standard tactic was treatment by siloplasty in the first few days after birth (Figures 1, 2). In this retrospective study, the results of siloplasty were analyzed using clinical data obtained over 6 years. Subsequently, the authors introduced a new method for treating gastroschisis—performing a one-stage reduction of intestinal loops after emptying the intestines through the rectum and the opened lumen of the appendix. Thus, 2 comparison groups were formed in which the effectiveness of the interventions was assessed for primary mortality and the presence of complications. As a result of the comparative analysis, the researchers came to the conclusion that in the group of one-stage reduction of eventrated organs, there was a lower mortality rate (5.7% vs. 35.7 in the siloplasty group), early adhesive obstruction occurred in 12.5% (vs. 28% in the siloplasty group), and there were no cases of sepsis and peritonitis (in contrast to the siloplasty group, in which peritonitis with sepsis developed in 22%) (55).

It is worth noting that the above study was conducted on a fairly small sample, in addition, there was a very careful selection of patients, and therefore it is quite difficult to extrapolate the results of this treatment to the general population.

Certainly, early surgical closure of the defect with all preventive measures to prevent compartment syndrome seems to be a completely rational technique. In addition, more thorough emptying of the intestinal loops allows, in comparison with the Bianchi procedure, to perform the intervention with a much more pronounced visceroabdominal disproportion. However, there is still a cohort of patients who require gradual reduction of the eventrated organs (79).

Thus, domestic authors proposed a new method of reduction of visceral organs, which resembles classical siloplasty, but has a number of advantages.

Firstly, a polymer bag of a fundamentally different shape is used—it is conical and narrows in the direction of connection with the edges of the abdominal wall defect. This bag is also sutured to the edges of the defect, after which it is vertically suspended, reducing the internal volume over several days and gradually reducing the eventrated organs (80).

Secondly, the development allows for multiple opening of the package, dissection of thread-like fibrin deposits, the possibility of selective immersion of visceral organs, removal of peritoneal effusion, manual expression of intestinal contents is described. This device provides much greater opportunities for monitoring and treating gastroschisis, and allows for planning additional interventions aimed at eliminating complications (39).

## Conclusion

Gastroschisis is a congenital pathology of the anterior abdominal wall, which is currently receiving special attention. Despite the fact that this defect is absolutely curable in most cases, a number of issues regarding safe surgical tactics, correct conservative therapy, optimal anesthetic care, and nutritional support have not yet been resolved (81).

Due to the fact that the incidence of gastroschisis tends to increase (in 1990, the incidence was at the level of 1:4,000, in 1996—1:2,900, in 2018—1:1,800), the heterogeneity of patients increases, individual phenotypes of gastroschisis and complications appear (53).

Particular attention should be paid to the cohort of patients with complicated gastroschisis. If previously it was believed that complications, after their resolution, do not affect the choice of surgical tactics, now there is a tendency to overestimate the risks of radical correction in patients with complicated disease (82).

In this regard, when choosing the optimal surgical intervention, preference should be given to those methods that make it possible to modify treatment in the event of complications (50).

If primary one-stage correction of gastroschisis is impossible with severe visceroabdominal disproportion, then the use of delayed radical correction methods is associated with an increased risk of complications. The most frequently used method of siloplasty is associated with a fairly high incidence of adhesive disease, moreover, in some cases it is not possible to reduce the defect in a sufficiently short time, and the time frame for the presence of the siloplasty bag should not exceed 5 days (83). In this regard, modified methods of siloplasty are being developed, in particular, the method proposed by domestic authors (Professor D.A. Plokhikh and co-authors). This method expands the scope of interventions performed during the period of reduction of eventrated organs, which probably increases freedom from complications. At the same time, it is necessary to collect remote treatment results and conduct randomized multicenter studies (47).

Techniques of delayed (up to several months) defect repair, used in cases of significant disproportion, are the only treatment option in some cases. Both suturing under a skin flap and patches made of various materials are used. These operations have the highest risk of complications, as they are often accompanied by tension of skin sutures, development of compartment syndrome, and infection of the skin suture (84, 85).

Indeed, the clinical results of gastroschisis treatment depend to the greatest extent on the extent of the abdominal wall defect. The more pronounced the visceral-abdominal disproportion, the longer it takes to correct the congenital defect. At the same time, the risks of complications and the likelihood of severe gastroschisis increase significantly (86).

The most reliable factor that can be relied on when predicting outcomes is intra-abdominal pressure. Other prognostically significant factors of complicated gastroschisis include fetal malnutrition, suboptimal nutritional support—feeding with mixtures with a high content of protein substrates that promote dysbiosis (87).

Finally, any treatment strategy for gastroschisis requires high intestinal lavage, installation of a gas-discharge tube, gastric lavage, and measures to prevent aspiration. Increased pressure in the lumen of hollow organs contributes to a decrease in perfusion of the wall of the hollow organ, which increases edema, contributes to the development of compartment syndrome, a decrease in the barrier function of the intestine, and the development of peritonitis (88).
